# Faith, fasting, and well-being: Emirates Neurology Society consensus guidelines on safe Ramadan fasting in Parkinson’s disease

**DOI:** 10.3389/fneur.2025.1720571

**Published:** 2025-12-11

**Authors:** Shivam Om Mittal, Pierre Krystkowiak, Carl Johan Ramberg, Pournamy Sarathchandran, Ali Hassan, Vittorio Iantorno, Mahesh Cirasanambati, Mohamed Al Aloma, Tanmoy Maiti, Suhail Al Rukn

**Affiliations:** 1 Parkinson’s Disease & Movement, Disorders Center, Cleveland Clinic, Abu Dhabi, United Arab Emirates; 2 Specialized Rehabilitation Hospital, Abu Dhabi, United Arab Emirates; 3 Sheikh Khalifa Medical City, Ajman, United Arab Emirates; 4 Department of Neurology, University Hospital Sharjah, Sharjah, United Arab Emirates; 5 Tawam Hospital and Sheikh Tahnoon Medical City, Al Ain, United Arab Emirates; 6 King’s College Hospital, Dubai, United Arab Emirates; 7 Burjeel Hospital, Abu Dhabi, United Arab Emirates; 8 Rashid Hospital, Dubai, United Arab Emirates; 9 Cleveland Clinic Abu Dhabi, Abu Dhabi, United Arab Emirates; 10 Rashid Hospital, UAE University, Dubai, United Arab Emirates

**Keywords:** Parkinson’s disease, Ramadan fasting, motor fluctuations, dopaminergic therapy, sleep disturbances

## Abstract

**Background:**

Parkinson’s disease (PD) is a progressive neurodegenerative disorder characterized by motor and non-motor symptoms. During Ramadan, fasting Muslims abstain from food, drink, and often medications between sunrise and sunset.

**Objective:**

To review the clinical considerations, therapeutic strategies, and cultural factors relevant to managing PD patients during Ramadan fasting, and to provide practical recommendations for neurologists and healthcare providers.

**Methods:**

This review synthesized existing guidelines (e.g., BIMA Ramadan Compendium), literature on drug pharmacokinetics during fasting, and clinical expertise from PD specialists in Middle Eastern and global Muslim populations. Discussions at a PD consensus meeting informed a stepwise algorithm for individualized care.

**Results:**

Pre-Ramadan risk assessment is essential, with stratification by disease stage. Early PD (Hoehn and Yahr stage 1–2) patients on monotherapy may fast safely with minimal adjustments, while moderate PD (Hoehn and Yahr stage 3) with multiple daily levodopa doses or combination therapy, requires consolidation of levodopa doses, addition of long-acting agents, and avoidance of dose stacking. Advanced PD patients who have troublesome motor/non-motor fluctuations and dyskinesias as well, and are taking medications multiple times per day are often unsuitable for fasting. Common complications include response fluctuations, dyskinesias, and sleep disturbances exacerbated by altered circadian rhythms. Long-acting dopaminergic therapies, including Dopamine Agonists (rotigotine patches and other extended-release (ER) oral agents), adjunctive agents (opicapone, rasagilline and safinamide), and Device-Aided Treatments (DAT; subcutaneous foslevodopa-foscarbidopa, subcutaneous continous subcutaneous apomorphine infusion, levodopa-carbidopa intestinal gel and deep brain stimulation) can help stabilize motor and non-motor fluctuations. Sleep hygiene measures and behavioral adjustments further support patient well-being. Cultural and spiritual motivations strongly influence adherence, requiring sensitive counseling and involvement of caregivers and religious leaders.

**Conclusion:**

Safe Ramadan fasting in PD requires comprehensive pre-Ramadan assessment, stage-specific therapeutic strategies, and proactive management of both motor and non-motor complications. Shared decision-making that integrates medical, psychological, and religious considerations is vital to optimize patient outcomes while respecting spiritual values.

## Introduction

Tremors, rigidity, bradykinesia, and postural instability are some of the symptoms of Parkinson’s disease, a neurodegenerative condition that mostly impairs motor function (1). Many Muslims who observe the holy month of Ramadan must contend with the difficulty of fasting for 29 to 30 days straight from sunrise to sunset. This difficulty is exacerbated for PD patients by the requirement to adhere to rigorous treatment schedules and the possibility of symptom swings brought on by extended fasting hours.

Assessing risk is crucial for PD patients who want to fast, according to guidelines like the British Islamic Medical Association’s (BIMA) Ramadan Compendium. Patients with advanced PD are more at risk since their symptoms fluctuate more and they are more likely to require multi-daily dose regimens. These people could require close supervision (2).

As far as we are aware, there are no published guidelines for the medical treatment of Parkinson’s disease (PD) during the month of Ramadan. There are effective therapies for Parkinson’s disease, and they often include multiple daily drug intakes. A significant withdrawal syndrome could result from stopping PD treatment, in addition to making symptoms worse.

Diabetes management during Ramadan offers a valuable parallel for chronic neurological disorders such as Parkinson’s disease. Both conditions require structured pre-Ramadan risk assessment, targeted patient education, and individualized medication adjustment to ensure safety during fasting. Diabetes care frameworks emphasize pre-fasting assessment, multidisciplinary planning, and continuous monitoring—principles that can be adapted to Parkinson’s disease to support shared decision-making and patient autonomy.

According to Kamel et al. (3), the June 2016 Ramadan fast in Kuwait was difficult because of the lengthy fasting hours (about 3:30 a.m.–7 p.m.) and the hot weather (between 25 and 50 °C). Nevertheless, a vast majority of patients were able to finish the entire month of fasting, even if they experienced mildly worsened PD symptoms and increased fatigue.

While some patients continued or decreased their antiparkinsonian therapy to one or two daily doses, about one-third of patients fasted rigorously without taking any medication. Reduced physical activity, improved medication absorption on an empty stomach, and overall Ramadan wellbeing were all factors that contributed to successful fasting. Although patients who quickly lowered their medication did not experience any severe side events, abrupt withdrawal entails a risk of neuroleptic malignant-like syndrome, which highlights the significance of progressive tapering under supervision.

Long-acting dopaminergic therapies, including Dopamine Agonists (rotigotine patches and other extended-release (ER) oral agents), adjunctive agents (opicapone, rasagilline and safinamide), and Device-Aided Treatments (DAT; subcutaneous foslevodopa-foscarbidopa, subcutaneous apomorphine infusion, levodopa-carbidopa intestinal gel and deep brain stimulation), according to the patient’s tolerance, are advised to promote safe fasting. The observational methodology and limited sample size of Kamel’s study were identified as drawbacks and larger cohorts of research are probably required to more precisely evaluate the feasibility and safety of fasting in Parkinson’s disease.

## Benefits of Ramadan in PD

While Ramadan fasting presents clear challenges for people with Parkinson’s disease (PD), emerging evidence suggests that it can offer several physiological, psychological, and spiritual benefits, many of which are relevant to the disease process and patient well-being (3, 4).

### Physiological benefits

Intermittent fasting is associated with improved insulin sensitivity, lipid balance, and reduced oxidative stress—factors that are important for vascular and brain health in PD (5). Studies in experimental models indicate that fasting may enhance neuroprotective pathways, such as autophagy and mitochondrial resilience, and upregulate brain-derived neurotrophic factor (BDNF) supporting neuronal health (6–8). Fasting may also improve gut-brain axis function, reduce inflammatory markers, and protect against dopaminergic neurodegeneration, which is central to PD pathology (4, 8). For patients who are overweight or experience medication-related weight gain, fasting can help normalize body weight; controlled eating windows may also improve gastrointestinal symptoms such as bloating and dyspepsia (5).

### Non-motor and psychological benefits

From a psychological perspective, many Muslims with PD report enhanced mental clarity, spiritual fulfillment, and emotional stability during Ramadan—effects that may help mitigate stress and improve non-motor symptoms like anxiety and depression (9, 10). Some patients experience improved mood and a subjective sense of well-being, facilitated by shared goals and religious engagement (9, 11). Adherence to structured sleep–wake and meal cycles during Ramadan may, in some cases, stabilize circadian rhythms and improve sleep patterns, though others with fragile sleep architecture may find this challenging (3).

### Spiritual and social benefits

Engagement in Ramadan fasting can provide a renewed sense of purpose, dignity, and social inclusion, with patients actively participating in religious and cultural practices (3, 10). Community support and shared rituals promote connectedness and mobilize family and caregiver involvement, potentially reducing stigma, isolation, and promoting better psychological coping with chronic illness (3, 11).

## Methods

This review was developed by synthesizing:

- Existing guidelines (e.g., BIMA, JAMA Neurology recommendations on fasting and neurological disease)
- Literature on drug pharmacokinetics during fasting.
- Clinical expertise in managing PD patients during Ramadan across Middle Eastern and global Muslim populations.

We propose a stepwise algorithm, integrating evidence and practical experience, to assist neurologists in tailoring therapy during Ramadan (Table 1).

The methods were discussed in the PD consensus meeting and reviewed by expert clinicians. Based on this, we propose a stepwise algorithm, integrating evidence and practical experience, to assist neurologists in tailoring therapy during Ramadan (Table 1).

**Table 1:** Stages of Parkinson’s disease and their implications for Ramadan fasting.

Stage	Clinical features	Functional status	Medication characteristics	Implications for Ramadan fasting
Early PD (typically first 3–5 years after diagnosis, Hoehn and Yahr stage 1–2)	Mild motor symptoms, often unilateral or mild bilateral involvement; little or no functional impairment	Independent in activities of daily living (ADLs)	Usually managed with monotherapy (levodopa or dopamine agonist); regimens are simple and low frequency	Most patients can safely fast with minor medication adjustments (e.g., switch to CR/ER formulations, once-daily adjuncts, or rotigotine patch)
Moderate PD (Hoehn and Yahr stage 3)	Bilateral motor involvement or midline symptoms (postural instability, gait disturbance); motor complications (wearing-off, dyskinesia) may emerge	Functional disability begins to impact daily activities; still partly or largely independent	Medication regimens more complex; often multiple agents with TID–QID levodopa dosing	Fasting feasible but requires careful restructuring with long-acting drugs, dose consolidation at Suhoor/Iftar, and adjuncts to minimize OFF periods
Advanced PD	Severe motor fluctuations, frequent OFF periods, disabling dyskinesias, falls; non-motor symptoms (cognitive impairment, psychosis, autonomic dysfunction) common	Increasing dependence in ADLs; may require assistance for most or all activities	Complex polypharmacy; device-aided therapies often required (Vyalev, apomorphine, LCIG, DBS)	Fasting generally not recommended; continuous infusion therapies cannot be interrupted; religious exemption (Fidya) should be considered

### Step 1: Pre-Ramadan assessment

**Figure 1:**
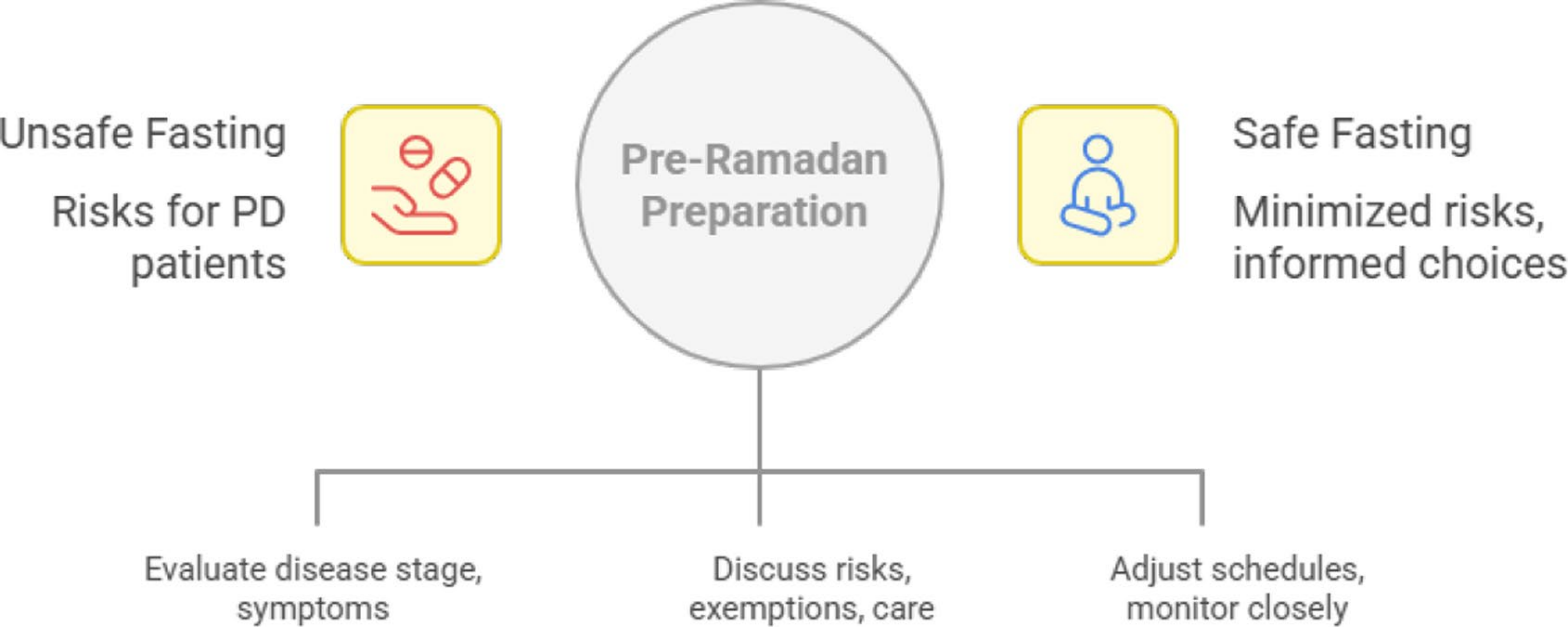
Safe Ramadan fasting: a preparation guide for Parkinson’s disease.

The pre-Ramadan period is critical for planning safe fasting in patients with Parkinson’s disease (PD) (Figure 1). Neurologists should adopt a structured approach that incorporates risk stratification, patient and family counseling, and medication review, ideally conducted 2–4 weeks before the start of Ramadan to allow for adjustments and monitoring.

#### Risk assessment

**Figure 2:**
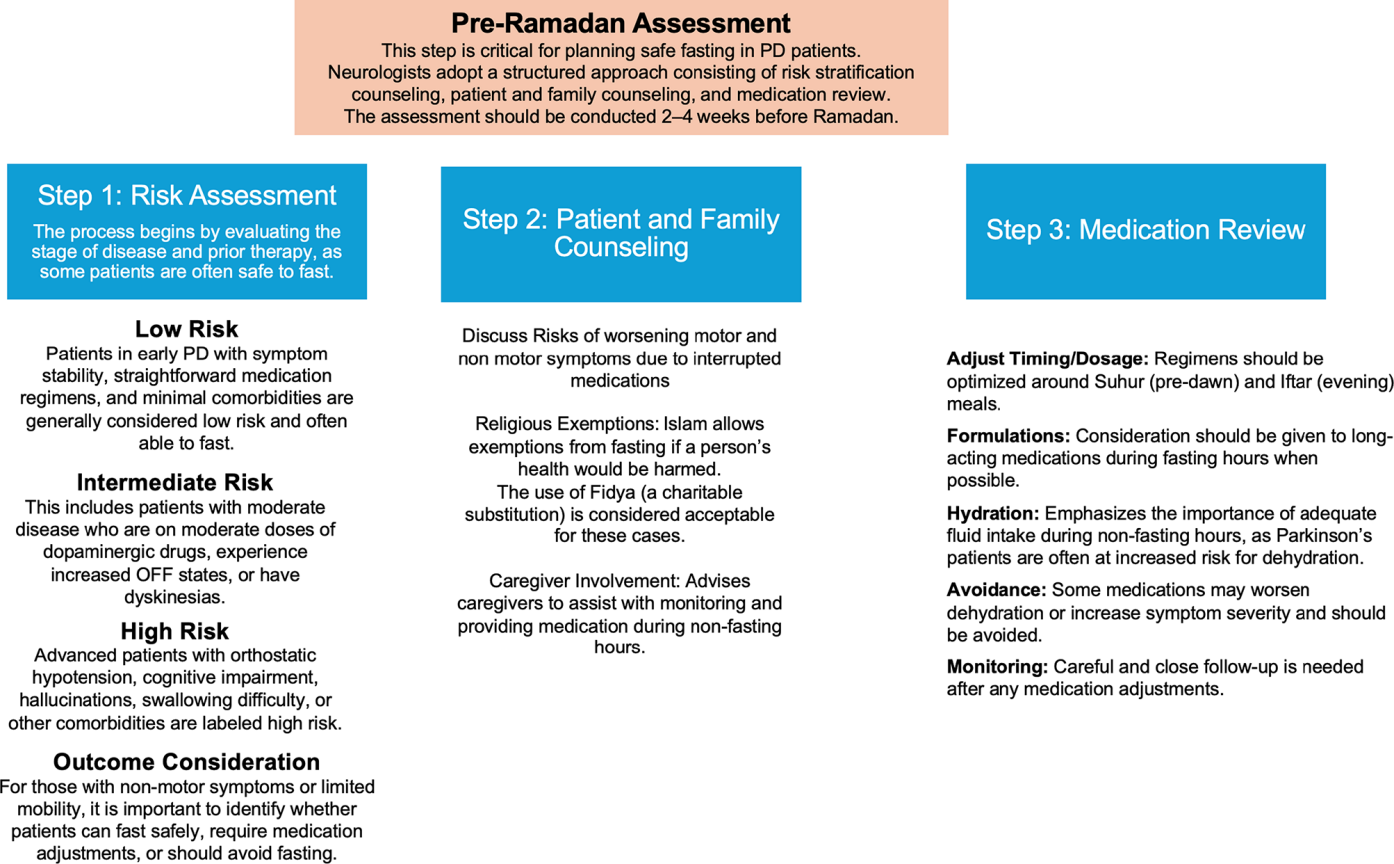
Three-step framework to guide safe fasting in Parkinson’s disease.

The first and most crucial step in preparing patients with PD for Ramadan is comprehensive risk assessment (Figure 2). This process involves evaluating the stage of disease, stability of symptoms, complexity of pharmacological regimens, and presence of comorbidities. Patients with early PD (Hoehn and Yahr stage 1), usually managed with simple monotherapy, are often considered low risk and may safely fast with minimal medication adjustments. Those in the moderate stage (Hoehn and Yahr stage 3), requiring multiple daily levodopa doses or combination therapy, carry an intermediate risk as fasting hours increase the likelihood of OFF states, dyskinesias, or nocturnal impairment. Patients with advanced PD, with troublesome motor and non-motor symptom fluctuations, are considered hight risk because interruption of therapy can lead to severe symptom rebound, functional decline, or even withdrawal syndromes. Patients who have device aided therapies such as deep brain stimulation (DBS), subcutaneous infusion therapies such as foslevodopa-foscarbidopa infusion, apomorphine infusion (CSAI) or levodopa-carbidopa intestinal gel (LCIG) could plan to do the fasting if the symptoms are under good control with device-aided therapies.

Beyond motor features, clinicians must also assess non-motor complications—such as orthostatic hypotension, cognitive impairment, hallucinations, and swallowing difficulties—which can be exacerbated by dehydration and irregular drug absorption during fasting. Risk assessment should therefore not be limited to motor disability alone but encompass the broader spectrum of disease burden, enabling neurologists to identify which patients can fast safely, which require strict modifications, and which should be advised against fasting altogether.

#### Patient and family counseling

Neurologists should provide individualized counseling sessions well before Ramadan (Figure 2).

- Discussion points:
  - Risks of worsening motor symptoms and complications if medication schedules are interrupted.
  - Religious exemptions: Islam permits non-fasting for patients whose health would be harmed. *Fidya* (charitable substitution) may be an acceptable alternative.
  - The importance of caregiver involvement in monitoring and medication administration during non-fasting hours.
  - Early recognition of red-flag symptoms: severe/prolonged OFF states, confusion, hallucinations, falls, or inability to swallow medications.
  - Fasting is not obligatory for patients with Parkinson’s disease who have dementia or psychosis, as these conditions may impair judgment and increase medical risk. Such patients should be advised to consult local religious authorities for guidance. Patients and caregivers should also be informed that in case of severe OFF states or medical complications during fasting hours, breaking the fast early is religiously permissible and medically advisable
- Communication strategies:
  - Use clear, non-technical language when addressing patients and families.
  - Involve local religious leaders or Imams when appropriate, to reinforce that exemptions are religiously valid and protect patient dignity.
  - Provide written instructions or Ramadan-specific medication schedules.

##### Clinical pearl

Counseling is most effective when the conversation acknowledges both the medical realities and the spiritual motivations of the patient, fostering shared decision-making rather than paternalistic advice.

### Step 2: management by disease stage

**Figure 3:**
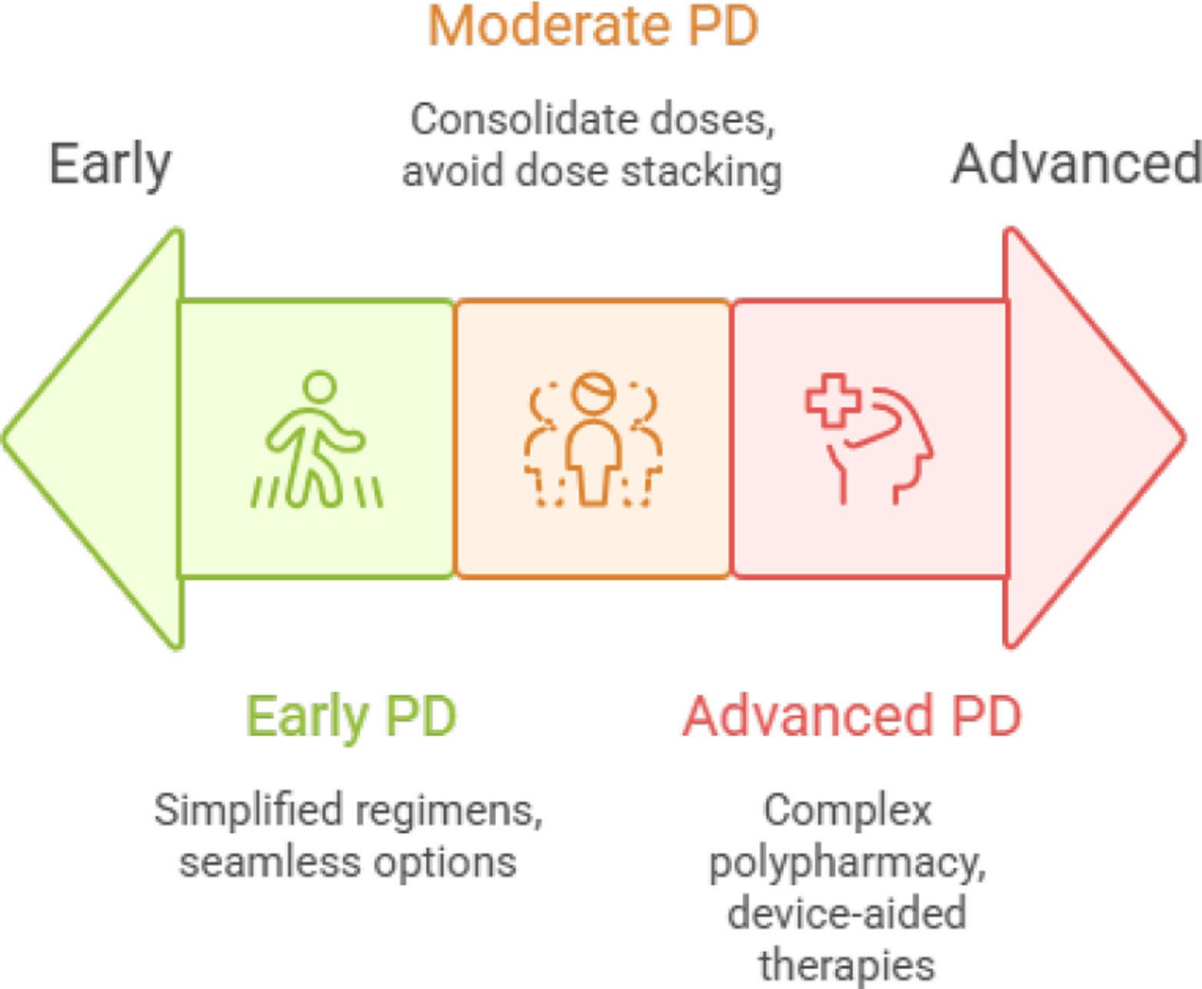
Disease stages in Parkinson’s and corresponding medication strategies.

Management of Parkinson’s disease (PD) during Ramadan must be individualized according to disease stage, symptom burden, and complexity of pharmacotherapy. The following stage-based approach offers practical strategies for neurologists (Figure 3).

#### Early Parkinson’s disease

##### General principle

Patients within the first 3–5 years of diagnosis usually have mild, unilateral or early bilateral motor symptoms with preserved independence in daily activities. Most are on monotherapy (levodopa or a dopamine agonist) or simple dual therapy. These regimens are generally easier to adapt to the restricted dosing opportunities of Ramadan (Suhoor—pre-dawn meal—and Iftar—evening meal).

**Figure 4:**
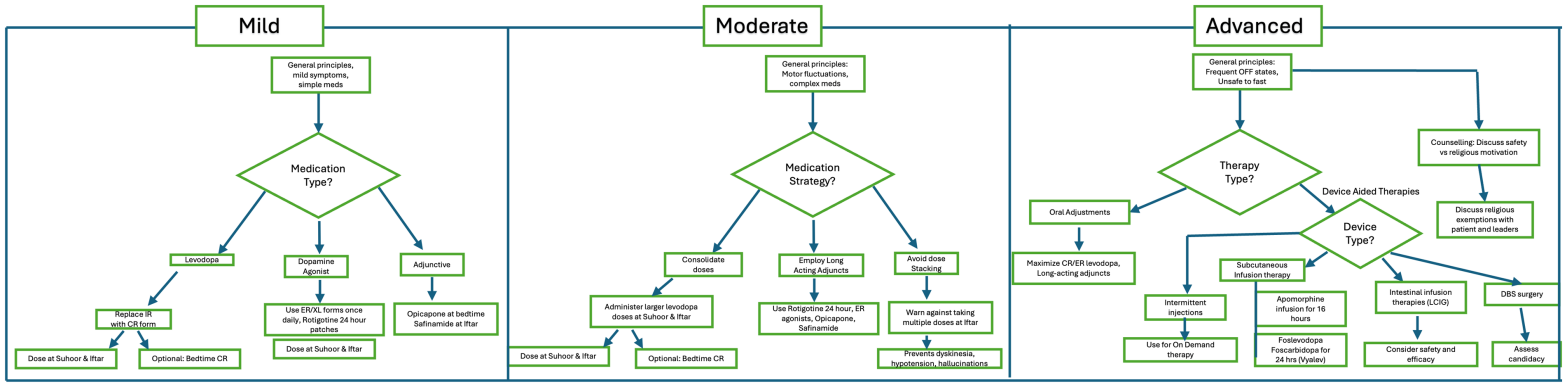
Clinical decision pathways for mild, moderate, and advanced Parkinson’s disease.

##### Medication strategies

- Levodopa:
  - Replace immediate-release (IR) forms with controlled-release (CR) or sustained-release (SR) formulations (e.g., *Sinemet CR, Madopar HBS*) to extend ON time through fasting hours.
  - Dosing at Suhoor and Iftar provides reasonable coverage, with a possible bedtime CR dose for nocturnal rigidity or akinesia.
- Oral Dopamine Agonists (DA):
  - *Pramipexole ER* or *ropinirole XL* can be administered once daily at either Suhoor or Iftar.
  - Their long half-life allows stable dopaminergic stimulation across fasting hours.
- DA in patch (rotigotine):
  - A transdermal, non-oral therapy that delivers continuous stimulation over 24 h.
  - Particularly valuable for patients struggling with medication timing or gastrointestinal absorption issues.
- Adjunctive Once-Daily Options:
  - *Opicapone*, a COMT inhibitor at bedtime to prolong levodopa action.
  - *Safinamide and Rasagilline*, MAO-B inhibitors, at Iftar or Suhoor for additional stabilization.

##### Clinical pearl

Most early PD patients tolerate simplified regimens well. Anticipate the possibility of morning OFF symptoms, especially if the Suhoor dose is delayed or missed. Rotigotine patches are often the most seamless option in this group.

#### Moderate Parkinson’s disease

##### General principle

As disease progresses, bilateral motor involvement and fluctuations emerge. These patients often require multiple daily doses of levodopa (TID–QID) and adjunct therapies. Fasting poses greater challenges because long intervals without medication predispose to OFF states and symptom breakthrough (Figure 4).

##### Challenges

- Long fasting hours may leave patients untreated during the middle of the day.
- Dose clustering around Iftar can provoke peak-dose dyskinesias.
- Symptoms’ unpredictability is common.

##### Medication strategies

- Consolidate Doses:
  - Administer larger doses of levodopa at Suhoor and Iftar, supplemented by a bedtime CR formulation for nocturnal coverage.
  - Care must be taken to avoid excessive single-dose exposure, which may worsen dyskinesia.
- Employ Long-Acting Adjuncts:
  - *Rotigotine patch* for continuous dopaminergic stimulation.
  - *Pramipexole ER* or *ropinirole XL* once daily for background coverage.
  - *Opicapone* at bedtime to reduce wearing-off.
  - *Safinamide* at Iftar and Rasagilline at Suhoor to smooth fluctuations and support ON time.
- Avoid Dose Stacking:
  - Warn against taking multiple levodopa doses in quick succession at Iftar. This increases the risk of dyskinesia and orthostatic hypotension post-meal.

##### Clinical pearl

Families often misinterpret peri-Iftar worsening as medication failure. These may be OFF symptoms due to the prolonged fast, not inadequate therapy. Conversely, sudden abnormal movements after Iftar often represent levodopa-induced dyskinesias. Differentiating the two helps in precise adjustment.

#### Advanced Parkinson’s disease

##### General principle

Advanced PD is marked by frequent OFF states, disabling dyskinesias, postural instability, falls, and significant non-motor features (cognitive impairment, psychosis, autonomic dysfunction). Complex polypharmacy or device-aided therapies are often required. Ramadan fasting is most difficult and often unsafe at this stage.

Subcutaneous infusion therapies such as foslevodopa–foscarbidopa and continuous subcutaneous apomorphine infusion represent valuable options for maintaining continuous dopaminergic stimulation during Ramadan; however, these therapies remain relatively new, high-cost, and not widely accessible due to limited regional availability and reimbursement constraints. Clinicians should therefore balance the potential benefits of these advanced treatments with practical feasibility and patient-specific resources when planning fasting regimens.

Patients with advanced Parkinson’s disease who also experience autonomic dysfunction—particularly neurogenic orthostatic hypotension (nOH)—require additional precautions. During fasting hours, the risk of dehydration and postural symptoms increases, which can precipitate falls, dizziness, or syncope. Non-pharmacologic strategies should be strongly encouraged, including maintaining adequate hydration during non-fasting hours, using compression stockings, elevating the head of the bed, and avoiding rapid postural changes.

In individuals with severe autonomic instability or those requiring frequent pharmacologic interventions such as midodrine, fludrocortisone, or droxidopa, fasting may be medically unsafe. In such situations, it is appropriate to advise patients about religious exemption through Fidya, in consultation with local religious authorities, to ensure both medical safety and spiritual well-being.

##### Medication and device strategies

- Oral Adjustments:
  - Maximize CR/ER levodopa formulations.
  - Continue rotigotine patch or ER dopamine agonists for continuous stimulation.
  - In selected patients, transitioning from oral to device-aided therapies (e.g., foslevodopa-foscarbidopa, or CSAI) during Ramadan may facilitate continuous dopaminergic coverage and simplify regimens, thereby improving safety and fasting tolerance.
- Device-Aided Therapies:
  - Subcutanous Infusion therapies:
    - Apomorphine injections and Pump:
      - Intermittent subcutaneous injections can rescue OFF states even during fasting hours, provided administration is tolerated and deemed religiously acceptable, as these injections do not involve oral intake.
      - CSAI therapy for 16 h (sometimes 24 h, which can be relevant during Ramadan) during the day (monotherapy is not usually possible in majority of the patients).
      - CSAI monotherapy is rarely feasible during Ramadan because it typically requires daytime administration with regular monitoring and supplemental oral doses for complete control. However, CSAI can be combined with long-acting dopaminergic preparations during non-fasting hours to maintain symptom stability while minimizing OFF periods
      - Continuous infusion may be religiously permissible, as it bypasses the oral route.
      - However, neurologists should encourage consultation with local Fatwa authorities.
    - Foslevodopa—foscarbidopa Infusion pump:
      - Delivered 24 h and monotherapy is highly possible.
      - Continuous infusion may be religiously permissible, as it bypasses the oral route.
      - However, neurologists should encourage consultation with local Fatwa authorities.
  - LCIG infusion (Levodopa-Carbidopa Intestinal Gel):
    - Requires uninterrupted 16–24 h delivery, most of the time in monotherapy.
    - Interruptions are dangerous, with risk of severe OFF or withdrawal.
    - Patients on LCIG are not suitable for fasting.
  - Deep Brain Stimulation (DBS):
    - Fasting is usually possible if oral regimens can be simplified into long-acting formulations.

##### Clinical pearl

Many advanced PD patients should not fast, but cultural and spiritual motivations may lead them to insist. In these cases, neurologists must provide compassionate yet firm counseling, balancing patient safety with respect for religious sensitivities. Religious exemptions (*Fidya*) should be discussed openly, ideally with involvement of religious leaders (see Supplementary material).

## Response fluctuations in Parkinson’s disease

A central challenge in Parkinson’s disease management is the occurrence of response fluctuations—motor and non-motor—particularly in patients receiving long-term levodopa therapy, which critically affect quality of life and increase treatment complexity (1, 12, 13). During Ramadan, when medication timing is constrained, these fluctuations can be amplified, making their recognition and management essential for patient safety (13, 14).

### Motor fluctuations

Short-duration fluctuations, such as freezing of gait or paradoxical kinesis, typically last seconds to minutes. Freezing restricted to OFF periods may be mitigated by increasing ON time but freezing occurring in both ON and OFF states is notoriously resistant to treatment (13, 14).

Medium-duration fluctuations include wearing-off and on–off phenomena, commonly arising after years of levodopa use. These lead to the re-emergence of parkinsonian symptoms or trigger dyskinesias when plasma drug levels fluctuate. Long-duration fluctuations reflect delayed adaptation to levodopa, sometimes persisting for up to 2 weeks, highlighting the importance of slow outpatient adjustments (12, 13).

Dyskinesias vary by phase: OFF-period dystonia (often painful), biphasic dyskinesias (severe, ballistic), and peak-dose choreiform or dystonic, usually painless dyskinesias (12). These can be particularly challenging when doses must be clustered around Suhoor and Iftar.

### Non-motor fluctuations

Non-motor fluctuations may manifest in the psychiatric and autonomic domains:

Psychiatric: panic, anxiety, depression, irritability (14).

Autonomic/physical: pain, bowel or bladder dysfunction, variable blood pressure, sweating, and respiratory irregularities (1, 12).

These symptoms reflect unstable or fluctuating dopaminergic stimulation and frequently parallel motor fluctuations. An apomorphine acute challenge may help clarify whether they emerge from under- or over-stimulation of dopamine receptors (12).

### Clinical relevance to Ramadan

Motor and non-motor fluctuations complicate fasting because restricted medication windows increase vulnerability to OFF states and dyskinesias (13, 14). For moderate-to-advanced patients, clustering doses at Iftar or Suhoor can trigger peak-dose dyskinesias, while long fasting intervals heighten risks of disabling OFF periods, anxiety, and autonomic instability. Understanding fluctuation patterns is fundamental when tailoring Ramadan regimens, with a focus on long-acting, continuous therapies (e.g., rotigotine patch, extended-release dopamine agonists, opicapone, safinamide, rasagilline) to minimize oscillations in dopaminergic tone (1, 12).

### Sleep and Parkinson’s disease during Ramadan

Sleep disturbances are among the most disabling non-motor symptoms of Parkinson’s disease (PD), affecting up to 60–80% of patients (15, 16). During Ramadan, altered circadian rhythms, late-night prayers (Taraweeh/Qiyam), and early waking for Suhoor disrupt sleep continuity and architecture, compounding existing sleep issues in PD (14). These changes may worsen vulnerability to fragmented sleep, REM sleep behavior disorder (RBD), insomnia, nocturnal akinesia, and excessive daytime sleepiness.

Common sleep problems in PD during Ramadan include insomnia and sleep fragmentation (from shortened sleep windows and late-night activity); intensified RBD due to night-time arousals and reduced REM sleep; more pronounced nocturnal akinesia with inadequate dopaminergic coverage; and excessive daytime sleepiness aggravated by sedating medications and lost nighttime rest (15, 16).

### Management strategies

Optimizing dopaminergic therapy is key: prescribing a bedtime dose of controlled/extended-release levodopa and considering a rotigotine patch for continuous 24-h coverage can minimize nocturnal OFF periods. Adjust timing of ER dopamine agonists (e.g., pramipexole at Iftar) to maximize nighttime efficacy while balancing risk of daytime somnolence (16, 17). Importantly, once daily opicapone has demonstrated improved sleep satisfaction and PDSS scores in studies such as BipARK and may be considered to reduce nocturnal motor symptoms (18).

Further management of sleep architecture includes split sleep schedules (partial rest after Iftar and after Suhoor), promoting a quiet, dark environment, and avoiding caffeine after Iftar or Taraweeh (16). For RBD, clonazepam or melatonin may help, though sedation must be monitored. Reducing nocturia by limiting evening fluids and supporting hydration earlier are also recommended, and sedating medications before Suhoor should be avoided to minimize risks of morning confusion or falls (15, 16).

Behavioral adjustments such as a short daytime nap (Qailulah) to mitigate sleep debt, and caregiver education on sleep hygiene and awareness of nocturnal confusion or hallucinations, are crucial (16).

In summary, sleep disturbances during Ramadan are multifactorial, arising from both disease-related neurobiology and religious lifestyle changes. Proactive management with tailored medication timing—including consideration of opicapone—rotigotine patch, and structured sleep hygiene can significantly improve nocturnal rest and reduce daytime somnolence in Parkinson’s disease (15, 17).

### Cultural and religious beliefs on fasting in elderly Parkinson’s patients

Spirituality and religious belief can be quite helpful in managing long-term conditions like Parkinson’s disease (PD). Spirituality has been demonstrated to assist patients cope with the symptoms and changes in lifestyle that come with chronic illness, as well as to preserve self-esteem and offer emotional support (19, 20). Decisions regarding fasting or other religious activities may be influenced by cultural and religious views for older PD patients. Adapting to changes brought on by illness frequently leads to existential contemplation, which may heighten one’s interest in or dependence on religion and spirituality (21).

It’s critical to distinguish between three types of religious changes in the context of Parkinson’s disease (PD): contingent reductions in religious practice brought on by cognitive, physical, or social challenges; and intrinsic changes in religiosity brought on by neural degeneration. Reactive responses to illness can either increase or decrease religious faith (22, 23). Despite the physical and cognitive difficulties of Parkinson’s disease, elderly persons may continue to observe fasting as part of their religious or cultural practice because of the rituals’ spiritual value.

## Conclusion

Fasting during Ramadan presents unique challenges for patients with Parkinson’s disease (PD), affecting both motor and non-motor symptoms due to altered medication schedules, prolonged fasting hours, and lifestyle changes. Evidence suggests that structured pre-Ramadan assessment, individualized risk stratification, and stage-specific medication adjustments are essential to minimize OFF periods, dyskinesias, and sleep disturbances (Table 2). Continuous or long-acting dopaminergic therapies, such as rotigotine patches, extended-release dopamine agonists, and adjunctive agents like opicapone or safinamide, can help stabilize motor function and reduce fluctuations. Non-motor symptoms, including sleep disruption and autonomic instability, require proactive behavioral and pharmacological interventions, while psychological, spiritual, and social factors influence patient adherence and well-being. Cultural and religious motivations may drive patients, particularly the elderly, to continue fasting despite medical risks, highlighting the importance of compassionate counseling and shared decision-making. Despite emerging observational data, there remains a paucity of large-scale, systematic studies evaluating the physiological and clinical effects of fasting in Parkinson’s disease and the optimal management strategies across disease stages.

**Table 2:** Summary of Ramadan fasting recommendations across Parkinson’s disease stages.

Step	Focus	Key recommendations	Clinical pearls
1. Pre-Ramadan Assessment	Risk evaluation & planning	Risk stratifies: Early, Moderate, Advanced PD Review medications Counsel patient & family (discuss exemptions / Fidya) Trial regimen adjustments 2–3 weeks before Ramadan	Risk assessment should consider motor and non-motor symptoms; not all patients are suitable for fasting
2. Early PD	Usually monotherapy, easier to adapt	Levodopa CR/SR at Suhoor & Iftar ER dopamine agonists (pramipexole ER, ropinirole XL) once daily Rotigotine patch (continuous 24 h) Adjuncts: Opicapone (bedtime), Safinamide (Iftar), Rasagilline (Suhoor)	Anticipate “morning OFF” symptoms; rotigotine patch provides best stability
3. Moderate PD	Multiple daily doses → risk of OFF states	Consolidate levodopa at Suhoor + Iftar + bedtime CR Rotigotine patch for continuous coverage ER dopamine agonists once daily Opicapone (bedtime), Safinamide (Iftar), Rasagilline (Suhoor) Avoid Iftar dose stacking	Differentiate peri-Iftar OFF (long fasting gap) from levodopa-induced dyskinesias
4. Advanced PD	Frequent OFFs, disabling dyskinesia, device dependence	Maximize CR/ER oral formulations + patches/ER agonists Foslevodopa-Carbidopa infusion therapy for 24 h is a good solution Apomorphine: PRN or continuous infusion (16–24 h), less likely as monotherapy in minority patients. LCIG- 16-24 h through PEG J tube → fasting not recommended DBS: possible if simplified regimen	Many advanced PD patients are unsuitable for fasting; emphasize safety and discuss exemptions (Fidya)
5. Monitoring During Ramadan	Follow-up & safety	Early review in first week, then as needed Monitor: dehydration, orthostatic hypotension, OFF states, dyskinesias, delirium, sleep issues Reinforce hydration/nutrition during non-fasting hours Engage caregivers & religious leaders	Close follow-up ensures early detection of complications; proactive caregiver involvement is vital
