# Supplementary figures and images for: Faith, fasting, and well-being: Emirates Neurology Society consensus guidelines on safe Ramadan fasting in Parkinson’s disease
Source: Front Neurol. 2025 Dec 11;16:1720571. doi: 10.3389/fneur.2025.1720571 (PMC12742308; doi:10.3389/fneur.2025.1720571)

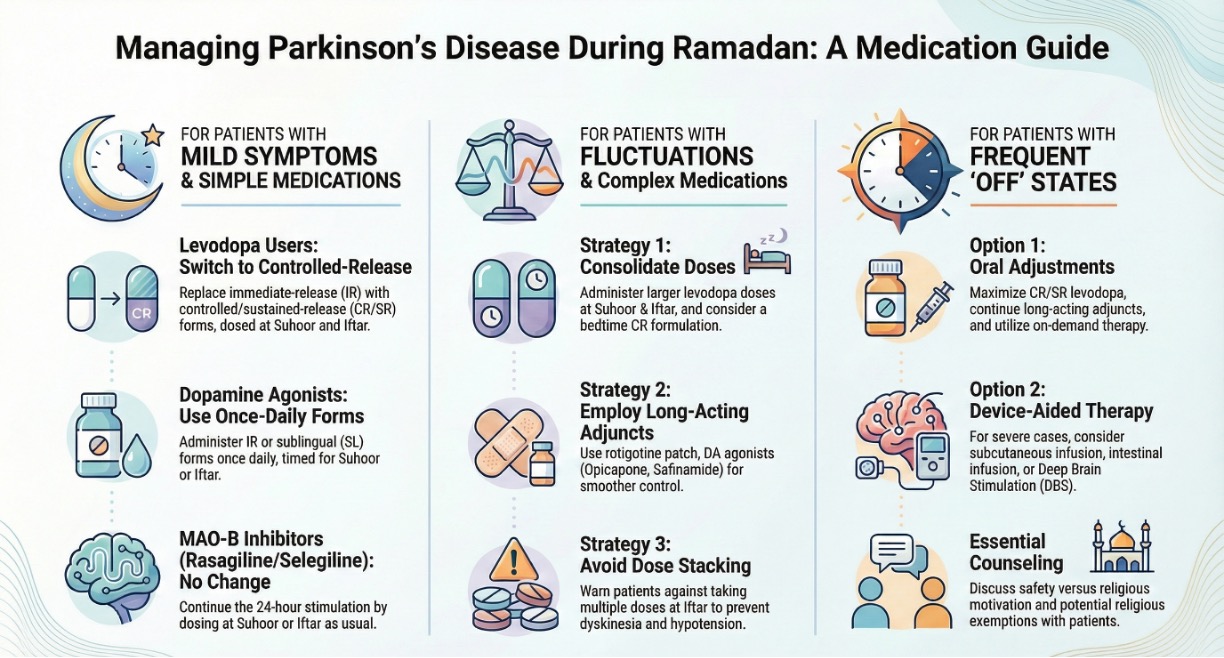

Supplement: Supplementary file 1 [file Image_1.jpg]
